# Variant calling in nonmodel organisms with snpArcher

**DOI:** 10.1093/molbev/msag220

**Published:** 2026-08-26

**Authors:** Cade Mirchandani, Abdelmajid Omarjee, Guillaume Achaz, Erik D Enbody, Gregg W C Thomas, Timothy B Sackton

**Affiliations:** Department of Biomolecular Engineering, University of California, Santa Cruz, Santa Cruz, CA 95064, USA; Stochastic Models for the Inference of Life Evolution (SMILE), CIRB, Collège de France, Université PSL, CNRS, INSERM, Paris 75005, France; Institut de Systématique Evolution Biodiversité, Muséum national d’Histoire naturelle, CNRS, SU, EPHE, UA, Paris 75005, France; Stochastic Models for the Inference of Life Evolution (SMILE), CIRB, Collège de France, Université PSL, CNRS, INSERM, Paris 75005, France; Department of Ecology and Evolution, Center for Interdisciplinary Research in Biology, Université Paris-Cité, Paris 75006, France; Department of Computational Biology, Cornell University, Ithaca, NY 14853, USA; Informatics Group, Harvard University, Cambridge, MA 02138, USA; Informatics Group, Harvard University, Cambridge, MA 02138, USA

**Keywords:** population genetics, nonmodel species, bioinformatics

## Abstract

Population genomic studies in nonmodel organisms increasingly depend on whole-genome resequencing, yet translating raw reads into reliable variant callsets remains a practical challenge due to the complexity of multistep bioinformatics pipelines and the absence of species-specific best practices. Here, we present a step-by-step protocol for snpArcher, a Snakemake-based workflow that takes raw sequencing reads and a reference genome as input and produces a filtered, joint-called Variant Call Format (VCF) file suitable for downstream population genomic analysis. We guide users through six phases: installation and environment setup, sample sheet creation, run configuration, execution on local or high-performance computing systems, quality control review using an interactive HTML dashboard, and downstream analysis, focusing on postprocessing and filtering. The quality control (QC) dashboard aggregates individual-level metrics including principal component analysis, relatedness estimation, depth-missingness diagnostics, and admixture analysis to help identify batch effects, contamination, cryptic relatedness, and outlier samples before downstream analysis. We demonstrate the impact of sequential filtering steps on the site frequency spectrum and demographic inference using a dataset of 137 burrowing owl (*Athene cunicularia*) genomes, showing how removal of low-coverage individuals, sex-linked scaffolds, and regions of excess heterozygosity eliminates artifacts that would otherwise bias inference of population size history. This protocol is intended as a practical companion to the original snpArcher publication, enabling researchers working with nonmodel organisms to produce and evaluate analysis-ready variant callsets in a reproducible manner.

## Introduction

Population genomic studies increasingly rely on whole-genome resequencing to investigate questions ranging from the genetic basis of adaptation to the demographic histories of natural populations. As high-quality reference genomes become available for a growing number of species through initiatives such as the Vertebrate Genomes Project (Rhie et al. 2021) and the Earth BioGenome Project (Lewin et al. 2022), opportunities for population-level resequencing in nonmodel organisms have expanded considerably. Yet translating raw sequencing reads into a reliable, analysis-ready variant callset remains a substantial practical barrier for many researchers. Variant calling pipelines involve numerous interdependent steps: read trimming, alignment, duplicate marking, individual variant calling, joint genotyping, and filtering; each with software dependencies and parameter choices that can meaningfully affect downstream results. For nonmodel species, the challenge is compounded by the absence of known variant sites, curated gene annotations, and the species-specific best practices that have been developed over years of effort in well-studied model systems (Van der Auwera et al. 2013).

We previously designed snpArcher (Mirchandani et al. 2024), a Snakemake (Mölder et al. 2021) workflow that encompasses all steps in a variant (or single nucleotide polymorphism [SNP]) calling pipeline and uses popular state-of-the-art tools for each step. The original description of snpArcher (Mirchandani et al. 2024) focused on the design of the workflow, its benchmarking, and its application to 26 public resequencing datasets from nonmammalian vertebrates. This Protocol is intended as a practical companion to that paper. Where the original publication describes what snpArcher does and why, the present article provides step-by-step instructions for how to use it. By following this Protocol, readers will be able to (i) configure and execute snpArcher to produce a joint-called, multisample variant callset from either local sequencing data or public Sequence Read Archive (SRA) accessions; (ii) generate and interpret the interactive QC dashboard to identify potential issues such as batch effects, cryptic relatedness, contamination, or outlier samples; and (iii) apply postprocessing filters and sample exclusions in a reproducible manner to prepare a clean VCF suitable for downstream population genomic analysis.

## Overview of the workflow

**Figure 1:**
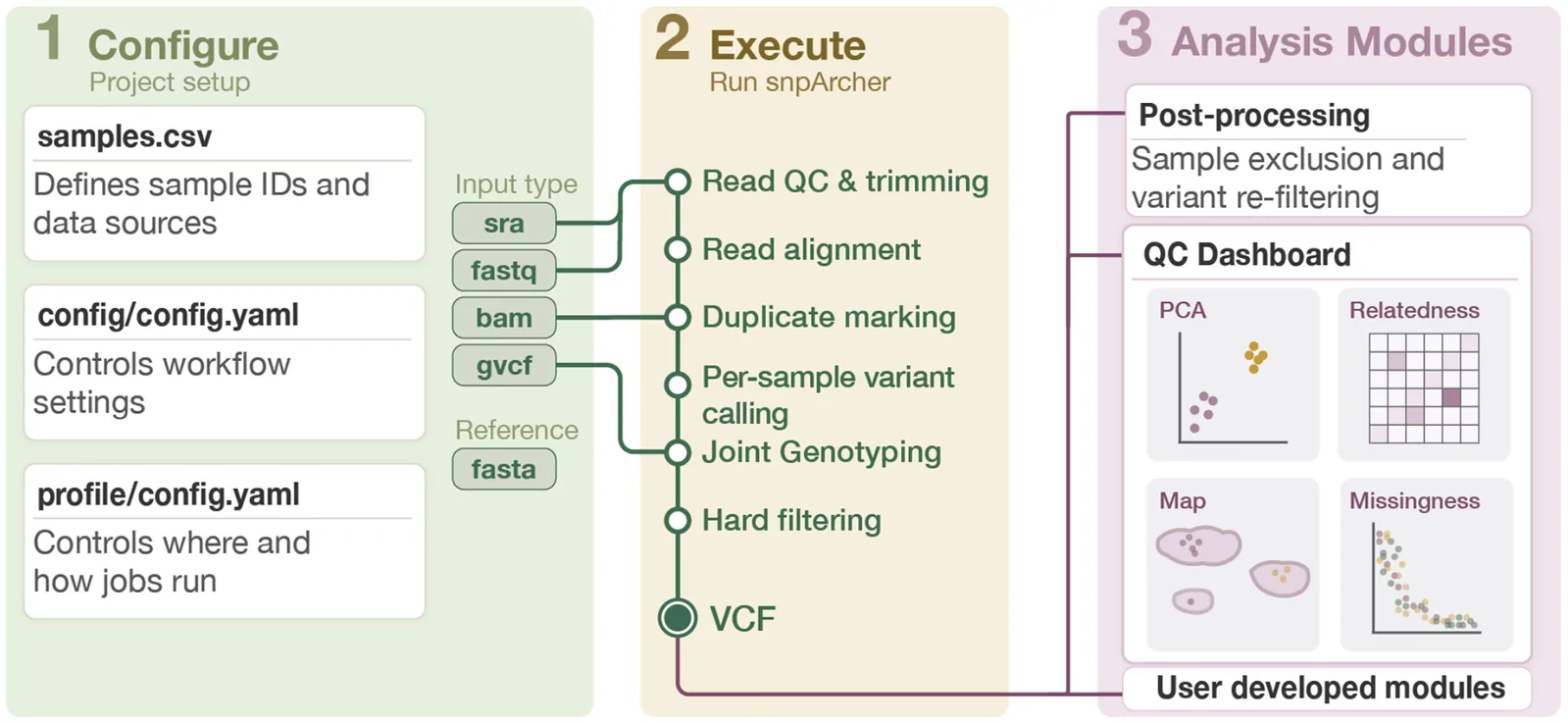
An overview of the snpArcher pipeline.

A standard snpArcher run starts with raw sequence reads and a reference genome assembly as input and produces a filtered, joint-called VCF, with optional modules that extend the core pipeline for quality control visualization, additional filtering, and downstream analysis (Fig. 1). Understanding the overall structure before diving into configuration details will help users anticipate the inputs required at each stage and interpret the outputs produced. A typical snpArcher analysis involves six phases: installation and environment setup, sample sheet creation, run configuration, execution, quality control review, and downstream analysis.

### Phase 1: installation and environment setup

snpArcher is distributed as a Snakemake (Mölder et al. 2021) workflow and uses Conda (https://anaconda.org/conda-forge) to manage software dependencies. As described in more detail below, users clone the snpArcher GitHub repository (https://github.com/harvardinformatics/snpArcher), install Snakemake in a dedicated environment, and configure parameters to control how and where each step of the pipeline runs. This phase also includes setting up the project directory structure, which snpArcher will populate with intermediate and final outputs.

### Phase 2: sample sheet creation

The sample sheet is a comma-separated file that defines the samples to be processed. Each row requires a sample identifier, an input type (eg “bam” or “sra”), and a local path or public accession pointing to the input data. An optional library identifier column supports workflows where samples have multiple sequencing runs that need to be merged.

### Phase 3: run configuration

A YAML configuration file controls workflow behavior for a particular dataset or run of snpArcher. Key settings include the path to the sample sheet, the reference genome (specified as a name and file path), variant calling parameters (tool selection, ploidy, heterozygosity prior), interval creation settings for parallelization, and toggles for optional modules such as QC, postprocessing, and trackhub generation. The configuration file also controls whether to compute callable sites based on coverage and mappability filters. For most datasets, the default settings provide a reasonable starting point, with adjustments needed primarily for ploidy, coverage thresholds, and module selection.

### Phase 4: execution

With the sample sheet and configuration file in place, the workflow is launched with a single Snakemake command. Snakemake handles dependency resolution, parallelization, and job submission via its executor plugin system. The core pipeline proceeds through data acquisition (downloading reads and reference genomes if accessions are provided), read preprocessing and adapter trimming (Chen et al. 2018), alignment with BWA-MEM v0.7.19 (Li 2013), duplicate marking (Tarasov et al. 2015), individual variant calling with GATK HaplotypeCaller v4.6.2, aggregation of per-sample calls into a GenomicsDB datastore, joint genotyping across all samples, and application of GATK hard filters (McKenna et al. 2010). Parallelization is achieved by splitting the reference genome into intervals at assembly gaps, allowing hundreds or thousands of concurrent jobs on well-provisioned clusters.

### Phase 5: quality control review

The QC module, enabled by default, produces an interactive HTML dashboard that aggregates individual-level metrics and population-level summaries. Users examine this dashboard to identify potential issues before committing to downstream analyses. Key visualizations include principal component analysis (PCA) to detect population structure or batch effects, a relatedness heatmap to flag close relatives or duplicate samples, and diagnostic plots relating sequencing depth, missingness, and heterozygosity. Outlier samples identified at this stage can be marked for exclusion in subsequent postprocessing.

### Phase 6: downstream analysis

After reviewing the QC dashboard, users may invoke additional modules depending on their analysis goals. In this Protocol, we demonstrate a workflow for postprocessing appropriate for population genetic analysis using the site frequency spectrum (SFS, see the heading “Postprocessing and Filtering”). This can be carried out with the built-in postprocessing module, which refilters the VCF after excluding flagged samples, applying user-configurable thresholds for mappability, coverage, minor allele frequency, and missingness. Users can also integrate their own Snakemake workflows as custom modules following the project's contribution guidelines.

## Before you begin

snpArcher requires Snakemake (version 9.0 or higher) and uses Conda or Mamba to manage software dependencies automatically. Users do not need to install individual bioinformatics tools (BWA, GATK, SAMtools, etc.) manually; Snakemake will create isolated environments for each workflow step on first execution and install the appropriate software within them. For cluster or cloud execution, the appropriate Snakemake executor plugin must be installed separately (eg snakemake-executor-plugin-slurm for SLURM-based systems; see https://github.com/snakemake/snakemake-executor-plugin-slurm for details).

### When is high-performance computing necessary?

For very small datasets, fewer than five samples at low coverage against a compact reference genome, a modern laptop or workstation with 16+ GB of RAM may suffice, primarily for testing or protocol familiarization. In practice, most analyses benefit substantially from high-performance computing (HPC) or cloud resources. Joint genotyping scales with both sample count and genome size, and the parallelization strategy in snpArcher is designed to exploit many concurrent jobs. We strongly recommend HPC or cloud execution for any dataset exceeding roughly ten samples, 10× average coverage, or a reference genome larger than 500 Mb.

### Estimating time and resources

Wall-clock time to completion depends primarily on three factors: genome size, number of samples, and sequencing depth. Larger genomes produce more calling intervals; more samples increase both the per-interval calling burden and the joint genotyping step; higher depth increases the computational cost of HaplotypeCaller per sample. The benchmarks in the original snpArcher publication provide concrete examples: a ten-sample, 10× coverage zebra finch dataset completed HaplotypeCaller in under 2 h of wall-clock time using the scatter-by-Ns parallelization strategy, compared to over 6 h using a traditional scatter-by-chromosome approach.

Memory requirements vary by workflow step. Alignment and variant calling are parallelized across many modest-memory jobs (4 to 8 GB per interval is typically sufficient). Joint genotyping with GenomicsDBImport can require more memory for large cohorts; 16 to 32 GB is a reasonable starting allocation, with adjustments if jobs fail. QC aggregation steps are generally lightweight. Intermediate files—particularly per-interval gVCFs—can accumulate rapidly and may require hundreds of gigabytes of scratch space for large datasets. Users on shared filesystems should also be mindful of I/O latency; locating temporary files on local scratch rather than networked project storage can substantially reduce bottlenecks.

## Project setup and configuration

### Phase 1: installation and environment setup

#### Installation

snpArcher requires Python, the conda package management software, and Snakemake to function, but otherwise can install all other dependencies. If you are working on an institutional cluster, conda may already be installed. Simply type conda --version to check: If you see the version number displayed, then it is installed. If you see a variation of the error message “command not found,” then conda is not already installed, and you will have to install it yourself. In that case, we recommend installing conda through Miniforge (https://github.com/conda-forge/miniforge). On Unix-like platforms (including Linux, MacOS, and Windows Subsystem for Linux), you can install the latest version of Miniforge appropriate for your system with these commands:

wget "https://github.com/conda-forge/miniforge/releases/latest/download/Miniforge3-$(uname)-$(uname -m).sh" bash Miniforge3-$(uname)-$(uname -m).sh

This will install the conda package management system. Once you have conda installed, you can create a new conda environment for your snpArcher projects in which you will install Snakemake:

conda create -c conda-forge -c bioconda -n \

snparcher "snakemake>=9"

In order to actually run snpArcher, you will need to activate the snparcher environment, eg by:

conda activate snparcher

Next, you will need to download the snpArcher pipeline itself. You can clone the repository directly from GitHub using git via:

git clone https://github.com/harvardinformatics/snparcher

You can also download a specific release by navigating to the Releases page at https://github.com/harvardinformatics/snpArcher/releases and downloading the assets for the version you wish to use.

#### Environment setup

We recommend organizing each snpArcher analysis as a single self-contained project directory. This makes it straightforward to archive, share, or revisit an analysis months later, because all configuration, metadata, and outputs are colocated. A clean starting layout looks like this:

my_project/

├── config/

│ └── config.yaml # workflow configuration

├── samples.csv # sample sheet

├── resources/

│ └── reference.fa # reference genome (if local)

└── results/ # created by snpArcher at runtime

The snpArcher repository that you cloned in the previous step is separate from your project directory. A single clone of the repository can serve multiple independent projects; you do not need to download snpArcher again for each new dataset. You can place the repository anywhere convenient on your filesystem—for example, in a central location such as ∼/software/snparcher/—and then point Snakemake to it using the --snakefile flag when launching the workflow:

snakemake \

--snakefile ∼/software/snparcher/workflow/Snakefile \

--use-conda \

--directory /path/to/my/project

Alternatively, if you prefer a fully self-contained setup, you can clone the repository directly into your project directory and run Snakemake from there. Either approach works; the key requirement is that the --directory flag points to your project directory (where config/config.yaml and samples.csv reside) and that Snakemake can locate the snpArcher Snakefile, either by running from within the repository or by specifying its path explicitly.

The results/ directory (and any scratch or temporary directories) will be populated by snpArcher during execution; users should not need to create subdirectories within it manually. If you are working on an HPC system, our general recommendation is that the project directory should reside on persistent project or group storage rather than scratch, so that configuration files and final outputs survive filesystem purge policies. However, specific cluster setups may see improved performance if run entirely on fast scratch storage. In that case, the project folder should be immediately copied back to persistent storage after snpArcher completes to avoid scratch purges.

### Phase 2: sample sheet creation

The sample sheet is a CSV file that tells snpArcher what samples to process and where their data are stored. Each row represents one sequencing run for one sample. Table 1 describes all recognized columns.

**Table 1:** Sample sheet columns.

Column	Required?	Description
sample_id	Yes	A unique sample identifier, for example, an NCBI BioSample ID. Must contain only alphanumeric characters, periods, underscores, and hyphens. The sample_id field will be used in the final VCF as the sample name, so it should be meaningfully linked to existing metadata or external databases.
input_type	Yes	One of fastq (local FASTQ files), srr (SRA run accession), bam (aligned BAM file), or gvcf (per-sample gVCF). Providing bam or gvcf inputs will launch the pipeline at the per-sample genotyping or the multisample genotyping steps, respectively. Multiple input types can be mixed on one sample sheet.
input	Yes	For fastq: forward and reverse read absolute paths joined with a semicolon (eg /data/sample_R1.fq.gz;/data/sample_R2.fq.gz). For srr: the SRA run accession (eg SRR12345678). For bam: absolute path to an aligned BAM file. For gvcf: absolute path to a per-sample gVCF file.
library_id	No	A library identifier. Defaults to sample_id if omitted. Used to control how BAMs from multiple rows are merged and how duplicate marking is applied (see below).
mark_duplicates	No	Boolean (true/false). Defaults to true. Set to false for amplicon or other protocols where duplicate marking is inappropriate.

#### Minimum viable sample sheet

At minimum, each row needs sample_id, input_type, and input. A two-sample sheet using SRA accessions:

sample_id,input_type,input

sample_A,srr,SRR12345678

sample_B,srr,SRR12345679

The same structure works for local FASTQ files, with forward and reverse read paths joined by a semicolon:

sample_id,input_type,input

sample_A,fastq,/data/sample_A_R1.fq.gz;/data/sample_A_R2.fq.gz

sample_B,fastq,/data/sample_B_R1.fq.gz;/data/sample_B_R2.fq.gz

#### Multirun and multilane samples

When a single sample has been sequenced across multiple runs or lanes, create one row per run, each with the same sample_id. snpArcher will align each run independently and then merge the resulting BAMs before variant calling. The library_id column controls duplicate-marking behavior during this merge: Rows sharing the same library_id are treated as technical replicates of one library (eg multiple lanes on the same flow cell), and duplicates are marked across the merged set. If a sample was prepared as two genuinely distinct libraries, assign them different library_id values, so that duplicate marking is performed within each library independently. The following example illustrates a multilane sample, a single-run sample, and mixed input types in one sample sheet:

sample_id,input_type,input,library_id

sample_A,fastq,/data/sample_A_lane1_R1.fq.gz;/data/sample_A_lane1_R2.fq.gz,lib1

sample_A,fastq,/data/sample_A_lane2_R1.fq.gz;/data/sample_A_lane2_R2.fq.gz,lib1

sample_B,fastq,/data/sample_B_R1.fq.gz;/data/sample_B_R2.fq.gz,lib2

sample_C,srr,SRR12345678,

### Phase 3: run configuration

Workflow behavior is controlled by a YAML configuration file located at config/config.yaml. This file governs the analytical decisions for a given run: which samples to process, which reference genome to use, variant calling parameters, and which optional modules to enable. A separate execution profile controls computational resource allocation and job submission; that file is covered in “Cluster Execution on HPC,” below.

#### Required fields

The following must be set for every run:

- **samples**: Path to the sample sheet CSV (eg samples.csv).
- **reference.name**: A short identifier for the reference genome (eg bTaeGut2). This string is used to name output directories and files.
- **reference.source**: The location of the reference FASTA. This can be a local file path, a URL, or a GenBank/RefSeq accession. If a URL or accession is provided, snpArcher will download the genome automatically.

samples: "samples.csv"

reference:

name: "my_organism"

source: "/data/reference/genome.fna.gz"

#### Variant calling settings

The variant_calling block controls tool selection and calling parameters:

- variant_calling.tool: The variant caller to use. Options are gatk (default), sentieon, bcftools, deepvariant, or parabricks. Each tool has its own sub-block of tool-specific settings; only the sub-block matching the selected tool needs to be configured. Sentieon requires a valid license, DeepVariant and Parabricks have their own hardware requirements (Graphics Processing Unit [GPU] access for Parabricks), and bcftools exposes mapping quality, base quality, and depth thresholds directly.
- variant_calling.ploidy: Integer ploidy for variant calling (default: 2).
- variant_calling.expected_coverage: Set to low or high to select appropriate internal parameter presets for the chosen caller. For most resequencing projects at 5× to 15× per sample, low is appropriate; high is intended for datasets with consistently deep coverage (eg >20×).
- variant_calling.gatk.het_prior: Prior expectation of per-site heterozygosity, used by GATK HaplotypeCaller. The GATK default (0.001) is calibrated for humans; many nonmodel organisms have substantially higher diversity, and adjusting this prior (eg to 0.005 or higher) can improve sensitivity for species with high nucleotide diversity.

variant_calling:

expected_coverage: "low"

tool: "gatk"

ploidy: 2

gatk:

het_prior: 0.005

#### Interval creation

snpArcher parallelizes variant calling by splitting the reference genome into intervals at runs of N characters (assembly gaps). The intervals.min_nmer parameter (default: 200) sets the minimum gap length used for splitting. Smaller values produce more, shorter intervals and greater parallelism, but also more overhead from job scheduling. For most vertebrate genomes, the default is a reasonable starting point.

#### Callable sites

For analyses that require distinguishing “no variant called” from “no data at this site”—such as estimating per-site heterozygosity or nucleotide diversity—enable the callable sites module. This module produces a BED file of genomic regions that pass both coverage and mappability filters, which can be intersected with the VCF to restrict analyses to reliably genotyped sites and to define positions where there is high confidence that the sample is homozygous for the reference allele. Coverage filtering removes regions where depth falls outside a user-defined range, with an auto option that calculates the mean coverage for each scaffold, and sets the minimum and maximum coverage thresholds to ½ and twice the mean, respectively. Mappability filtering removes regions where short reads cannot map uniquely.

callable_sites:

 generate_bed_file: true

 coverage:

  enabled: true

  fraction: 1.0

  min_coverage: auto

  max_coverage: auto

  merge_distance: 100

 mappability:

  enabled: true

  kmer: 150

  min_score: 1

  merge_distance: 100

#### Module toggles

Optional modules are enabled or disabled under the modules block. Each module has its own sub-block of settings that are relevant only when the module is enabled.

- qc: The interactive QC dashboard (recommended). The clusters parameter sets the number of k-means clusters for PCA visualization (default: 3). min_depth sets the minimum average depth for a sample to be included in the QC report (default: 2). An optional google_api_key enables satellite basemap rendering for the sample location map. exclude_scaffolds allows specific scaffolds to be omitted from QC calculations.
- postprocess: Sample exclusion and site-level refiltering. contig_size sets a minimum scaffold length to retain (default: 10000). maf, missingness, and exclude_scaffolds control minor allele frequency filtering, maximum missingness, and scaffold exclusions, respectively.

modules:

qc:

enabled: true

clusters: 3

min_depth: 2

google_api_key: ""

exclude_scaffolds: ""

postprocess:

enabled: false

filtering:

contig_size: 10000

maf: 0.01

missingness: 0.75

exclude_scaffolds: "mtDNA,Y"

### Inputs checklist

Before launching snpArcher, confirm that all required inputs are in place and accessible from the compute environment where the workflow will run.

Required:

(i) **Sequencing data**—one of the following input types (mixed inputs across samples are supported):
  - paired-end FASTQ files
  - aligned BAM files
  - per-sample gVCF files
  - SRA accessions
(ii) Reference genome—specified in config/config.yaml via reference.source. This can be a local FASTA path, a URL, or a GenBank/RefSeq accession; snpArcher will download and index the genome automatically if a remote source is provided. The genome should be the assembly you intend to use for all downstream analyses; switching references later requires rerunning the pipeline from alignment onward.
(iii) Sample sheet—a CSV file with at least sample_id, input_type, and input columns, validated per the guidance in “Phase 2: Sample Sheet Creation.”
(iv) Configuration file—config/config.yaml with samples, reference.name, and reference.source set, plus any nondefault variant calling or module settings (“Phase 3: Run Configuration”).

Optional:

(v) **Sample-level** metadata—geographic coordinates (latitude/longitude) are used by the QC dashboard to produce a sample map. Population labels or sample-type designations may be required by specific downstream modules (eg postprocessing exclusions). In the current version, these metadata are supplied through module-specific configuration or auxiliary files rather than the core sample sheet, keeping the manifest minimal and separating workflow metadata (what snpArcher needs to run) from sample metadata (what downstream analyses need to interpret results). Users of earlier versions of snpArcher (v1) who are accustomed to including columns such as SampleType, lat, and long directly in the sample sheet should consult the migration guide for updated conventions.

### Phase 4: execution

#### Sanity check with a test dataset

Before running snpArcher on your own data, we recommend confirming that the installation is working with the bundled example dataset. From the snpArcher repository root:

snakemake --use-conda --cores 4 \

--directory example/ \

--workflow-profile workflow-profiles/default/

This runs the core pipeline on five simulated samples against a small reference genome. The run should complete in ∼5 min on a machine with four cores. A successful run will produce output files under example/results/, including the filtered VCF and per-sample BAMs, without any error messages.

#### Dry-run for validation

Before committing compute resources to a full run, perform a dry-run to verify that snpArcher has parsed your configuration correctly and that the execution plan looks as expected:

snakemake --use-conda --dry-run \

--directory /path/to/my_project \

--workflow-profile workflow-profiles/default/

The dry-run does not execute any jobs. Instead, it resolves the full dependency graph (DAG) and reports which rules will be run, how many jobs are planned, and what output files will be produced. Check for the following:

- **Sample sheet parsed correctly:** Sample identifiers in the job list should match your sample_id values.
- **Expected rules are queued:** If you enabled the QC module, you should see QC aggregation rules in the plan; if you left postprocessing disabled, those rules should be absent.
- **Output paths match your intended layout:** Output files should appear under the results/ directory within your project.

#### Local execution

For small test datasets or compact analyses, conda-prefix flag to a central location can be run locally on a single machine without a job scheduler. Before running any Snakemake commands, make sure the snpArcher conda environment is active:

conda activate snparcher

All Snakemake commands shown below should be run from the snpArcher repository directory (the cloned snparcher/ folder), with the --directory flag pointing to your project directory. The canonical command for local execution is:

snakemake --use-conda --cores \

--directory /path/to/my_project \

--workflow-profile workflow-profiles/default/

The --use-conda flag tells Snakemake to automatically create and manage isolated conda environments for each step of the pipeline, installing the required bioinformatics tools (BWA, GATK, SAMtools, etc.) on first execution. This means users do not need to install these tools manually—Snakemake handles it. These environments are cached after initial creation, so subsequent runs will not reinstall software.

##### Conda environment storage

By default, Snakemake creates its per-step conda environments inside the project directory (under .snakemake/conda/). This means that each project will install its own independent copy of every tool, which can consume substantial disk space and time. To share environments across projects, set the --conda-prefix flag to a central location:

snakemake --use-conda \

--conda-prefix ∼/snparcher-envs \

--cores <N> \

--directory /path/to/my_project/ \

--workflow-profile workflow-profiles/default/

This tells Snakemake to create and cache all conda environments in ∼/snparcher-envs/ (or any path you choose). Any subsequent snpArcher run that uses the same --conda-prefix will reuse the existing environments rather than rebuilding them. We recommend setting this to a location on persistent storage with adequate quota. On shared clusters, a common choice is a directory under your group's project space.

The --cores flag specifies the maximum number of CPU cores Snakemake may use concurrently. Setting this to the number of available cores (eg --cores 4 on a laptop, --cores 16 on a workstation) allows Snakemake to parallelize independent steps of the workflow, such as running HaplotypeCaller on multiple intervals simultaneously. If --cores is set to 1, jobs will execute sequentially.

##### Monitoring progress

During local execution, Snakemake prints progress directly to the terminal, reporting which rules are starting, finishing, or failing. In addition, each step of the variant calling pipeline writes its own log file under logs/<step_name>/ within the project directory (eg logs/bwa_mem/, logs/gatk_haplotypecaller/). If a job fails, the terminal output will indicate which rule encountered the error; the corresponding log file will contain the full standard output and standard error from that command, which is usually sufficient to diagnose the problem.

##### Output locations

All workflow outputs are written to the results/ directory within your project. The core pipeline always produces a joint-called VCF (results/vcfs/raw.vcf.gz), a hard-filtered VCF (results/vcfs/filtered.vcf.gz), per-sample gVCFs (results/gvcfs/), per-sample BAMs (results/bams/), and a QC metrics summary (results/qc_metrics/qc_report.tsv). When optional modules are enabled, their outputs appear in dedicated subdirectories: the QC dashboard at results/qc/, callable sites files at results/callable_sites/, and postprocessed VCFs at results/postprocess/. Table 2 describes all main output files.

**Table 2:** Main output files produced by snpArcher.

Output	Path	Description	Condition
Raw VCF	results/vcfs/raw.vcf.gz	Joint-called VCF across all samples, before any filtering.	Always produced
Filtered VCF	results/vcfs/filtered.vcf.gz	VCF after applying GATK VariantFiltration hard filters. Filter annotations are added to the FILTER column; sites are retained but tagged as passing or failing.	Always produced
Per-sample gVCFs	results/gvcfs/{sample}.g.vcf.gz	Individual gVCF files for each sample. Useful for adding samples to future joint-calling runs.	Always produced
Per-sample BAMs	results/bams/markdup/{sample}.bam	Final aligned BAM with duplicates marked. Path is results/bams/merged/{sample}.bam when mark_duplicates is set to false.	Always produced
QC metrics	results/qc_metrics/qc_report.tsv	Per-sample summary statistics including mapping rate and mean depth.	Always produced
Callable sites (coverage)	results/callable_sites/coverage.bed	BED file of regions passing coverage filters.	callable_sites.coverage.enabled is true
Callable sites (mappability)	results/callable_sites/mappability.bed	BED file of regions passing uniqueness-of-mapping filters.	callable_sites.mappability.enabled is true
QC dashboard	results/qc/qc_dashboard.html	Interactive HTML quality control report with PCA, relatedness, depth, and admixture visualizations.	modules.qc.enabled is true
Postprocessed VCF	results/postprocess/filtered.vcf.gz	VCF after removing excluded samples and sites failing filters or with ref = N or AF = 0.	modules.postprocess.enabled is true
Clean SNPs	results/postprocess/clean_snps.vcf.gz	Biallelic SNPs from the postprocessed VCF after applying missingness, MAF, callable-site, and scaffold-exclusion filters.	modules.postprocess.enabled is true
Clean indels	results/postprocess/clean_indels.vcf.gz	Indels from the postprocessed VCF after applying the same filters.	modules.postprocess.enabled is true

Core pipeline outputs are always generated; optional outputs are produced only when the corresponding module or configuration option is enabled.

##### Practical considerations

Local execution is best suited for familiarization with the workflow, running the bundled test dataset, or processing a small number of low-coverage samples against a compact reference genome. Because all jobs compete for the same machine's memory and I/O bandwidth, performance degrades as sample count or genome size increases. If HaplotypeCaller or GenomicsDBImport jobs fail with out-of-memory errors on a local machine, reducing --cores to limit the number of concurrent memory-intensive jobs can help, though for datasets of any appreciable size, cluster execution is strongly preferred.

##### Long-running local jobs

Even on a workstation, a full snpArcher run may take hours or days. To prevent the workflow from terminating when a terminal session closes, run the command inside a terminal multiplexer such as screen or tmux:

tmux new -s snparcher

conda activate snparcher

snakemake --use-conda \

--conda-prefix ∼/snparcher-envs \

--cores 8 \

--directory /path/to/my_project \

--workflow-profile workflow-profiles/default/

# Detach with Ctrl-b, then d; reattach later with: tmux attach -t snparcher

#### Cluster execution on HPC

In most instances, one will want to run snpArcher on a HPC cluster, which will distribute individual parts of the workflow to compute nodes on the cluster with more resources than are available on personal computers. Since snpArcher is a Snakemake workflow, we rely on Snakemake's executor plugins to interface between the workflow and the cluster. This involves several steps: (i) installing the cluster executor plugin, (ii) setting up a workflow profile (.yaml file) to specify resources for each step of the workflow, and (iii) running the workflow in a way that will persist after you have logged off of your cluster.

First, you will need to determine what job scheduler (software that allocates resources on HPC clusters) your cluster uses. Snakemake supports several job schedulers with their executor plugins (https://snakemake.github.io/snakemake-plugin-catalog/index.html), and we will use SLURM as an example in this protocol since it is fairly common and snpArcher has been well tested on it. Once you know your job scheduler, find the corresponding executor plugin in the catalog. In the same environment in which you have Snakemake installed, install the executor plugin with either pip or conda/mamba using the commands provided in the plugin's documentation.

Next, you will need to set up a workflow profile, which is an additional YAML formatted configuration file, located at workflow-profiles/config.yaml, relative to your project root directory. In this file, you can set resources such as memory, time, and threads for each step of the workflow. We provide a default profile with sensible resource allocations for many datasets at snpArcher/workflow-profiles/default/config.yaml, so you should copy this file directly to the workflow-profiles/ sub-folder of your project directory.

mkdir -p/path/to/my_project/workflow-profile cp /path/to/snparcher/workflow-profiles/default/config.yaml \

/path/to/my_project/workflow-profiles/config.yaml

Then edit the copied profile to match your cluster. For SLURM, uncomment and set the slurm_partition and slurm_account (if required by your cluster) fields near the top of the file. If you use a different job scheduler, replace these with the equivalent fields for your executor plugin (see the plugin's documentation).

##### Temporary directory configuration

Many HPC clusters allocate only a small local /tmp partition on compute nodes. Several steps in the snpArcher pipeline, particularly GATK's GenomicsDBImport and BAM sorting, write substantial temporary data that can easily exceed this space, causing jobs to fail. To avoid this, set default-resources.tmpdir in your workflow profile to a directory on a fast networked scratch filesystem with adequate space:

default-resources:

tmpdir: "/scratch/$USER/tmp"

Ensure this directory exists before launching the workflow (mkdir -p /scratch/$USER/tmp). Ideally, the scratch filesystem should be high-performance and local to the cluster's compute nodes; avoid pointing it at slow NFS-mounted home directories.

With the profile in place, verify the configuration with a dry run:

snakemake --executor slurm --use-conda \

--conda-prefix ∼/snparcher-envs \

--dry-run \

--directory /path/to/my_project \

--workflow-profile /path/to/my_project/workflow-profiles/config.yaml

The --executor slurm flag tells Snakemake to submit individual steps of the workflow as jobs to the cluster rather than running them on the login node.

However, if you just run this command from the cluster's login node, the command would be interrupted anytime you disconnect from the cluster. Since most workflows will take hours to days to run, it is not practical to assume one could remain connected to the cluster the entire time. Instead, we recommend submitting the Snakemake command itself as a job to your cluster if your cluster setup allows running jobs to submit other jobs (eg as is common with SLURM). For cluster setups or queue management software where this is not possible (eg PBS), we recommend submitting jobs via tmux or screen. This workflow job will then distribute the other jobs to the cluster as well, and will remain running when you disconnect from the cluster. For SLURM, this means either submitting the Snakemake command directly to the cluster with srun, or within a job script with sbatch.

#!/bin/bash

#SBATCH --job-name=snparcher

#SBATCH --time=7-00:00:00

#SBATCH --mem=4G

#SBATCH --cpus-per-task=1

#SBATCH --output=snparcher_controller_%j.log

conda activate snparcher

snakemake --executor slurm --use-conda \

--conda-prefix ∼/snparcher-envs \

--directory /path/to/my_project \

--workflow-profile /path/to/my_project/workflow-profiles/config.yaml

The controller job needs minimal resources—it only orchestrates other jobs—so 4 GB of memory and a single CPU is typically sufficient. Set the time limit generously to ensure the controller remains active for the duration of the run.

While running on the cluster, it can be confusing to track the progress of Snakemake and the individual jobs that are run because several log files are generated. Table 3 summarizes where to find each type of log and what it contains.

**Table 3:** Log file locations and their contents.

Log file source	Location	Description	Context
Snakemake	.snakemake/logs/ <timestamp>.snakemake.log	Log produced by the main Snakemake process. Tracks overall pipeline progress, rule execution order, and errors. Also records the cluster job ID for each submitted job.	Local and cluster
Cluster	.snakemake/slurm_logs/<rule_name>/ <wildcard>/<SLURM_job_id>.log	Log produced by the cluster job scheduler (eg SLURM). Records resource-related errors such as out-of-memory or time-limit failures.	Cluster only
Individual job	logs/<rule_name>/<sample_id>/<run_id>.txt	Log produced by an individual rule invocation for a particular sample (eg logs/bwa_mem/, logs/gatk_haplotypecaller/). Contains standard output and standard error from the underlying bioinformatics tool.	Local and cluster

All paths are relative to the project directory.

In case of errors, we recommend checking these logs in the following order. First, consult the Snakemake log (.snakemake/logs/) to identify which rule failed and find the cluster job ID. Next, check the cluster log (eg .snakemake/slurm_logs/) for resource-related failures such as out-of-memory or time-limit errors. Finally, check the individual job log under logs/ for tool-specific error messages—for example, logs/bwa_mem/ for alignment errors or logs/gatk_haplotypecaller/ for variant calling errors. This last log is usually the most informative for diagnosing problems with the underlying bioinformatics command.

### Phase 5: quality control review

A key component of SNP calling is to evaluate the success of the variant calling algorithm. Biases in variant calling can occur from sequencing depth variation, read mapping success, reference genome bias, population structure, nucleotide diversity, and genome complexity, among other things. Rather than requiring the user to calculate summaries to evaluate these issues independently, snpArcher creates a report that provides visualizations to evaluate if there were any issues in the variant calling run. Note that the report focuses on “individual” metrics (eg average depth per individual) rather than “site-level” (eg Hardy–Weinberg equilibrium at one position) specific metrics; see the Postprocessing and Filtering section below for further discussion of site-level filtering. We made this decision, because issues with site-level quality will likely manifest as individual-level biases. Nevertheless, we recommend considering which site-level filters to apply in the postprocessing module, which can be partially informed from the QC module. For example, one could decide on a minor allele frequency filter after deciding how many individuals to retain after reviewing the QC report.

#### How to use the QC report

The QC report is provided as an HTML file, which can be opened in any modern web browser. The HTML dashboard uses Plotly widgets, meaning users can zoom, pan, and hover over points to see sample IDs. The report contains two high-level summaries at the top of the page. On the left, a summary of the snpArcher run and input data for the QC report is provided. The report itself is based on a random, linkage disequilibrium (LD)-pruned selection of 100k SNPs, after the removal of indels, nonbiallelic SNPs, sites with a minor allele frequency (MAF) <0.01, sites with >75% missing data, and samples <2× depth. This reduced VCF file is available in the QC directory.

The header of the report includes a summary of how many individuals were run through snpArcher, the number of SNPs detected, an approximation of nucleotide diversity (Watterson's theta [Watterson 1975]), and the average genome-wide depth (based on “read depth”). Nucleotide diversity is calculated after applying light filtering to the data, using Watterson's estimator. This value may differ from a user estimation of nucleotide diversity, for example, if different site-based filters are applied, or if you are interested in a different estimator of nucleotide diversity. This is a good example of why the QC report should be used to guide decisions about how to proceed with analyses, rather than relying on it for final interpretations. We define important QC terms in Box 1.

#### QC report figures

The report contains ten figures that can be used to guide decisions about which individuals to drop, how to apply site-level filters, and to determine if any biases in the dataset might influence downstream results. Below, we summarize how these figures can be used, which is based on investigating 142 snpArcher runs for the California Conservation Genomics Project (Shaffer et al. 2022). For this project, each run refers to a single species and ∼150 samples sequenced at 10× depth against a ∼2 Gb chromosome-scale genome.

##### QC Panel 1

**Figure 2:**
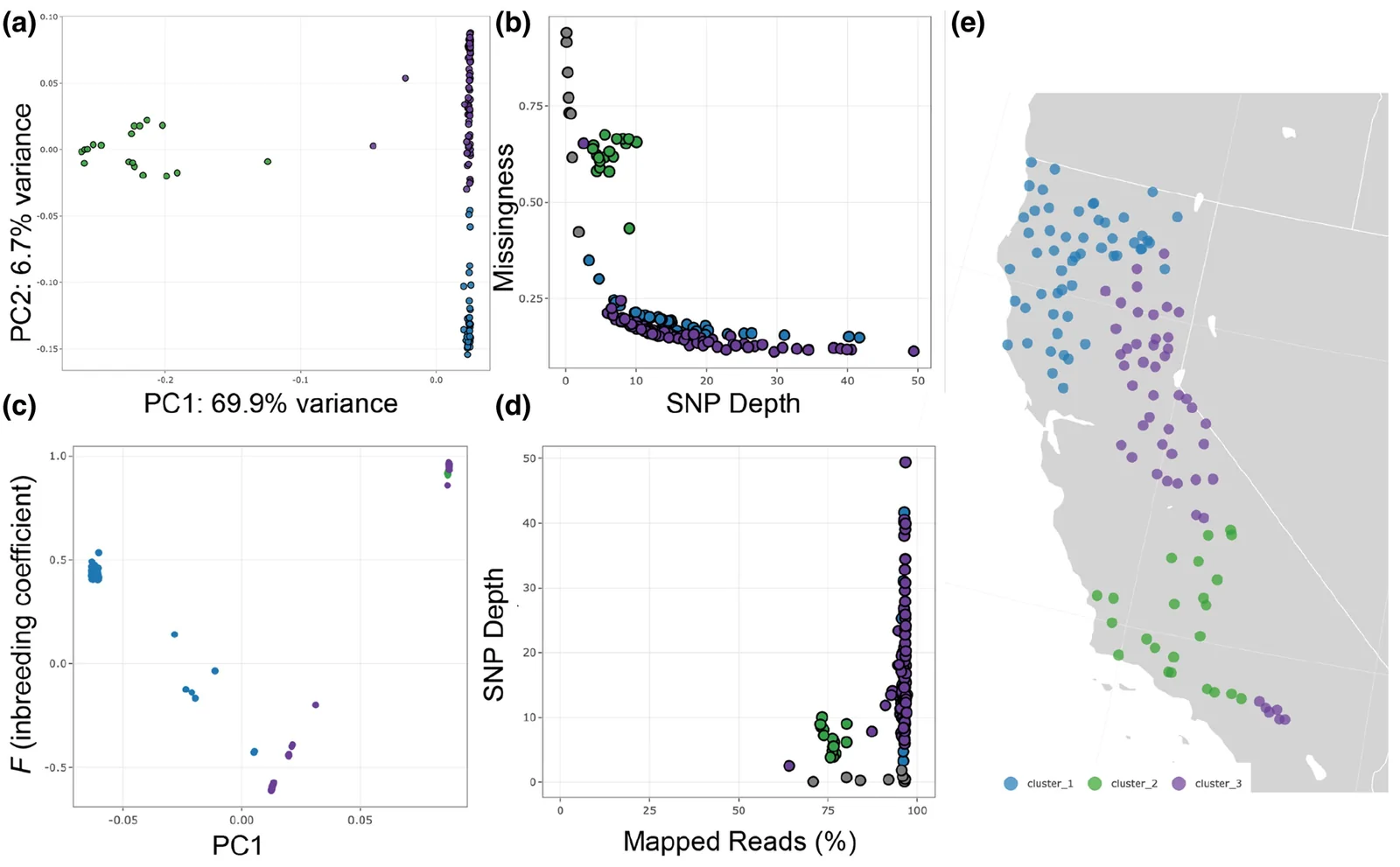
Example QC plots for various California taxa. a) Genomic PCA demonstrating substantial population structure in the output of a snpArcher run. Samples colored by K = 3. Note high percent variation explained by PC1, likely suggesting species-level divergence. b) SNP depth compared with fraction of sites with missing data for the same species as a). Note the cluster of samples that have a large portion of missing data, relative to depth, which is an indication of reference bias. These samples are likely divergent from the reference genome. c) Inbreeding coefficient against PC1, showing intermediate PC1 samples have an excess of heterozygotes (negative *F*). These individuals are likely hybrids. d) SNP depth against mapped reads for the same species as in a) and b). Note the cluster of samples with a low mapping rate. e) A map showing the distribution of PCA clusters: Most samples occur near other samples from the same cluster. An exception is found in the south, which may be worth investigating.

A PCA calculated using PLINK2 (Chang et al. 2015) with samples colored by k-means cluster assignments (default k = 3). The number of clusters can be modified in the configuration file, though we find k = 3 provides a reasonable approximation for populations that are expected to be relatively homogeneous. The rationale for this approach is that any grouping identified by the clustering algorithm could reflect underlying population structure or, alternatively, some technical bias in the data requiring further investigation. Percent variance explained is calculated relative to the first ten principal components. See example in Fig. 2a.

##### QC Panel 2

Sequencing depth against principal components 1 through 10. In our experience, a common cause of bias in low- to moderate-coverage sequencing datasets is sequencing depth variation among samples. When depth varies substantially across individuals, this technical artifact can manifest as a pattern captured by principal components, potentially confounding biological signals with technical noise. If a strong correlation exists between depth and one of the top principal components (PCs), this would indicate that bias in sequencing depth is greater than other sources of variation among individuals in the dataset. Nevertheless, sometimes there is so little structure in a dataset that the only variation observed is driven by depth-related biases in the data, eg the reduced efficacy of calling heterozygotes in low-coverage samples (Lou et al. 2021). Users should carefully examine this relationship and consider whether depth filtering or normalization is warranted prior to downstream analysis.

##### QC Panel 3

Comparison of mean SNP depth with SNP missingness for each individual, serving multiple diagnostic purposes. It provides a rapid visualization of the skew in sequencing depth among individuals and can also reveal evidence of reference bias. For example, if one of the clusters identified in the PCA exhibits systematically higher missingness at a given depth, this pattern may indicate that reference bias is contributing to observed structure in the data. Additionally, this plot is useful for determining how many individuals would be excluded under different sequencing depth thresholds, allowing users to make informed decisions about sample filtering. See example in Fig. 2b.

##### QC Panel 4

The relationship between mapping rate and sequencing depth. The mapping rate is calculated as the proportion of reads that successfully align to the reference genome. A lower mapping rate may indicate the presence of contaminants in the sequencing data or that the sample originates from a species other than the intended target. For example, a mapping rate below 80% suggests either that the sample is derived from a different species (resulting in many unmapped reads) or that ∼20% of the sequencing data originates from another source, such as bacterial contamination. See example in Fig. 2d.

##### QC Panel 5

Estimates of the inbreeding coefficient (F) for each individual. Values approaching +1 indicate extensive homozygosity, while values approaching −1 indicate an excess of heterozygotes relative to Hardy–Weinberg expectations. Users should examine samples that appear as outliers in the PCA, particularly those exhibiting strongly negative F values, as this pattern can indicate cross-contamination between samples during library preparation or sequencing. Note that population structure in the dataset will influence F, which can often be observed by the coloring from the PCA clustering. See example in Fig. 2c.

##### QC Panel 6

A neighbor-joining tree (Saitou and Nei 1987) constructed from a simple pairwise genetic distance matrix calculated among all samples in the dataset. Terminal branches are colored according to the k-means cluster assignments from the PCA, allowing users to assess concordance between clustering results and phylogenetic relationships.

##### QC Panel 7

A heatmap of the KING kinship coefficient (Manichaikul et al. 2010) matrix calculated using PLINK, which is used to evaluate whether closely related individuals are present in the dataset. Samples with kinship coefficients of ∼0.5 are effectively identical (or monozygotic twins), values near 0.25 indicate first-degree relatives (parent-offspring or full siblings), and values near 0.125 indicate second-degree relatives (half-siblings, grandparent-grandchild, or avuncular relationships). Users may wish to remove samples with kinship coefficients exceeding 0.354 to exclude very closely related individuals that can bias population genomic estimates such as effective population size and nucleotide diversity.

##### QC Panels 8 and 9

If the user provides geographic coordinates for each sample in the input files, QC Panel 8 displays a simple map with sample locations colored by the k-means cluster assignments from the PCA. If the user additionally provides a Google Maps Application Programming Interface (API) key in the configuration file, QC Panel 9 renders the same sample locations on a satellite basemap displaying topographic variation. Instructions for obtaining a Google Maps API key are available in the snpArcher documentation. See example in Fig. 2e.

##### QC Panel 10

Results from ADMIXTURE (Alexander et al. 2009) analysis run on the dataset for k = 2 and k = 3 ancestral populations. These values are arbitrarily selected, and no cross-validation is performed to determine the optimal number of clusters. Because the ADMIXTURE groupings are generated independently from the PCA clusters, samples in this figure are colored by their ADMIXTURE ancestry assignments rather than the k-means cluster colors used elsewhere in the report. This figure provides a simple visualization of model-based population structure, but formal analyses with appropriate cross-validation should accompany any publication using these data.

Box 1. Glossary of terms **ADMIXTURE:** A model-based clustering program that estimates individual ancestry proportions from multilocus SNP genotype data. Unlike PCA, ADMIXTURE assumes individuals are drawn from K discrete ancestral populations and estimates the proportion of each individual's genome derived from each population.

**Biallelic SNP:** An SNP where exactly two alleles are observed in the sample. Nonbiallelic sites (those with three or more alleles) are typically removed from population genomic analyses because they can arise from sequencing errors or paralogous mapping.

**GATK best practices filters:** A set of hard filters recommended by the Genome Analysis Toolkit in situations where variant quality score recalibration is not possible (Van der Auwera et al. 2013). These filters remove variants that fail quality thresholds for parameters such as strand bias, mapping quality, read position bias, and quality by depth.

**Indel:** An insertion or deletion mutation relative to the reference genome. Indels are typically removed from SNP-based analyses because they are more prone to be the result of alignment errors and can complicate downstream analyses.

**Inbreeding coefficient (F):** A measure of the probability that two alleles at a locus are identical by descent. Values range from −1 to +1, where positive values indicate excess homozygosity (inbreeding), zero indicates Hardy–Weinberg equilibrium, and negative values indicate excess heterozygosity (potentially indicating contamination or outbreeding).

**k-means clustering:** An unsupervised machine learning algorithm that partitions samples into k groups by minimizing within-cluster variance. In the QC report, k-means is applied to PCA coordinates to identify discrete sample groupings that may reflect population structure or technical artifacts.

**KING kinship coefficient:** A robust measure of pairwise relatedness calculated using the KING algorithm implemented in PLINK. Expected values are ∼0.5 for identical samples or monozygotic twins, 0.25 for first-degree relatives (parent-offspring, full siblings), 0.125 for second-degree relatives (half-siblings, grandparent-grandchild, avuncular), and 0.0625 for first cousins.

**LD-pruning:** The process of thinning SNPs to a subset of approximately independent markers to reduce biases caused by linkage disequilibrium. In the QC report, this is achieved by randomly retaining one SNP per genomic window, where the window size is calculated to yield ∼100,000 SNPs.

**Mapping rate:** The proportion of sequencing reads that successfully align to the reference genome. Low mapping rates (<80%) may indicate sample contamination, misidentification of species, or poor reference genome quality.

**Mean depth:** Average sequencing coverage across all individuals, estimated from read depth using the program mosdepth. Differs from variant depth, which is calculated only at polymorphic sites and does not account for invariant (monomorphic) positions.

**MAF:** The frequency of the less common allele at a biallelic locus in the sample. SNPs with very low MAF (eg <0.01) are often filtered because they may represent sequencing errors or provide limited information for population-level analyses.

**Missingness:** The proportion of genotype calls that are missing (not successfully called) for a given individual or SNP. High missingness can indicate poor sample quality, low sequencing depth, or systematic technical problems.

**Neighbor-joining:** A distance-based phylogenetic method that constructs an unrooted tree by iteratively joining the pair of operational taxonomic units that minimizes total branch length. In the QC report, it provides a rapid visualization of genetic relationships among samples.

**Nucleotide diversity (Watterson's θ):** A measure of genetic diversity estimated as the total number of segregating sites divided by the harmonic number (the sum of 1/i for i from 1 to n−1, where n is the sample size), scaled by genome length. This estimator is relatively insensitive to allele frequencies and provides an estimate of the population mutation rate (4Neμ).

**PCA:** A dimensionality reduction technique that identifies axes of maximum genetic variation in multivariate data. In population genomics, PCA of genotype data reveals population structure, with samples from the same population typically clustering together along the principal component axes.

**Reference bias:** A systematic tendency for reads carrying the reference allele to map more successfully than reads carrying alternate alleles, particularly when samples are genetically divergent from the reference genome. In this case, reads with true variants may not map. This can manifest as elevated missingness or reduced heterozygosity in divergent samples.

**Sequencing coverage (or depth):** The average number of times each nucleotide position in the genome is represented by aligned sequencing reads. Higher depth generally improves genotype calling accuracy, while low depth (<5×) can lead to reduced heterozygous calls and missing data.

**SNP:** A single base pair position in the genome where different alleles exist among individuals in a population. SNPs are the most common type of genetic variation and form the basis for most population genomic analyses.

### Postprocessing and filtering

In many real-world datasets, the raw variant calls output by snpArcher contain sample quality issues, mapping artifacts, and other biases that must be filtered prior to analysis. To guide users, we outline the impact of both individual- and site-level filtering on the SFS calculated from an example dataset of 137 diploid genomes of *Athene cunicularia* (burrowing owl) mapped on a scaffold-level genome assembly (Mueller et al. 2018). The SFS is a useful metric for assessing site-level quality issues.

There are several features of our example dataset that are relevant. First, there is heterogeneous sequencing depth across individuals, leading to a variable proportion of missing data. Second, the reference genome is typical of high-quality draft assemblies, with many chromosomes represented by a small number of large fragments. To demonstrate the impact of various filtering choices, we computed SFS using “deminfhelper,” released as part of “popsize” (Forest et al. 2024). We applied several sequential filtering steps, generating a series of four VCF files, all generated with “bcftools” (Danecek et al. 2021).

(i) Raw snpArcher output. In most cases, we recommend some degree of additional filtering to account for individual- and site-level noise, which is common in most sequencing experiments. For illustrative purposes, we first show the results computed from the VCF with only standard GATK hard filters applied, containing 137 diploid genomes.
(ii) Remove low-quality individuals. A common first step in filtering pipelines is to remove low-quality individuals. A small number of individuals with particularly low coverage or high missingness can contribute a large fraction of the errors. Individual quality can be assessed with the QC HTML produced by snpArcher. For example, if a few individuals appear to be outliers with low sequencing depth and high missingness (eg on QC Panel 3), it is often best to remove these individuals. Here, we identify average sequencing depth less than 8× as a useful threshold and remove 18 individuals below this depth. This can be done via bcftools directly (see below), or by setting the individuals to exclude and running the postprocess module.

bcftools view -S keep_samples.txt \

Athene_cunicularia_raw.vcf.gz -Oz \

-o Athene_cunicularia_cov8.vcf.gz

In this command, keep_samples.txt is a list of individuals with sequencing depth >8× (the individuals to keep); Athene_cunicularia_raw.vcf.gz is the input file name; Athene_cunicularia_cov8.vcf.gz is the output file name; and -Oz outputs compressed vcf.

(iii) Remove sex chromosomes. For many downstream analyses for many species, the hemizygous nature of sex chromosomes in the heterogametic sex poses challenges, as the number of haploid chromosomes varies between males and females. For simplicity, it often makes sense to remove the sex chromosomes. This can be done directly with bcftools, as shown below, or by setting the “contigs_to_exclude” parameter in the “postprocess” module. In our example data, we remove the five scaffolds that form the Z chromosome; the W chromosome is not assembled in this reference genome, and so we do not need to remove it here.

bcftools view -t \

^QEEU01000009.1,QEEU01000013.1,QEEU01000020.1,QEEU01000033.1,QEEU01000054.1 \

-Oz -o Athene_cunicularia_noZ.vcf.gz \

Athene_cunicularia_cov8.vcf.gz

In this command, the scaffolds after the “-t” option are the ones that are removed; the “^” before the list turns “-t” from “keep” to “remove.” We use the output from the previous filtering step as the input file and generate a new output file, Athene_cunicularia_noZ.vcf.gz; as before -Oz outputs compressed vcf.

(iv) *Filter low-quality sites.* Sequencing, assembly, and mapping artifacts can create variable-quality SNP calls across the genome. Duplicated or repetitive regions that are collapsed in the assembly can create regions of excess coverage and heterozygosity; a peak in the SFS at half the number of haploid genomes is typical of this failure mode. Misassemblies, mapping artifacts, or sequencing biases can also create regions of poor genotyping quality. For applications sensitive to these issues, such as SFS analysis, we recommend filtering both putative duplicated/repetitive regions and poorly genotyping regions.

The callable sites module in snpArcher will generate a BED file of callable regions, filtering out both regions of low coverage and regions of excess coverage; this can be used to filter the output VCF. In the absence of a callable sites BED, one can be approximated by using a combination of high heterozygosity filters and low SNP density filters. Given either a callable sites bed file or a bed file containing regions to filter, a final VCF can be produced with this code:

bcftools view -T ^Ac_high_het.bed \

Athene_cunicularia_noZ.vcf.gz \

-Oz -o Athene_cunicularia_noZ_filtered.vcf.gz

Here, we created a filter bed file using a sliding window of 100 single nucleotide variants (SNVs) and filtered out windows where (i) the average heterozygosity is more than twice the median value (a signal of potential unidentified duplicates) and (ii) the window is in the 5% of the windows with the lowest SNV density (a signal of poorly genotyped regions). For a callable sites bed file, use -T without the “^”.

Importantly, filtering steps (iii) and (iv) also change the effective length of the genome, since it is important to only consider the length of the genome that has been properly genotyped when normalizing analyses by site or computing statistics in sliding windows. With a callable sites bed file, one can compute the effective genome length simply as the sum of the callable intervals; with a filter bed file, the effective length is the genome size minus the sum of the filtered intervals. If the filtered or callable intervals are estimated based on a VCF only, we recommend estimating an effective length by computing the remaining genome length after filtering (L_VCF_), and L′, defined as L_VCF_ S/S_TOTAL_ (where S is the number of variants that were genotyped for all individuals—no missing data—and S_TOTAL_ is the total number of variants in the VCF file, including the ones with missing data). L′ measures the length of the genome that has been properly genotyped.

**Figure 3:**
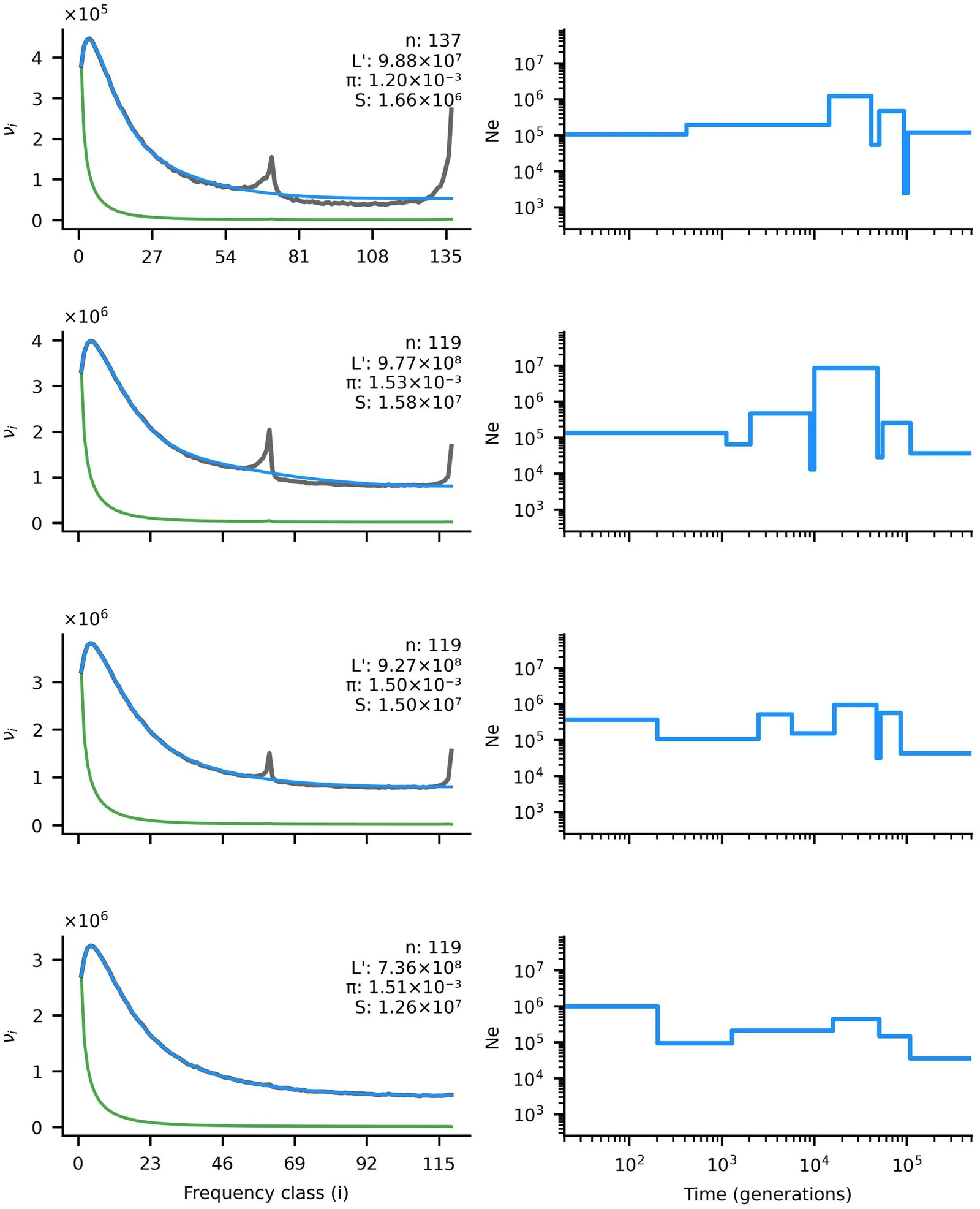
Left panels display three complementary SFS representations: the raw folded SFS (green), the transformed folded SFS (black), and the transformed SFS corresponding to the scenario fitted by *blockbuster* (blue). The corresponding right panel represents the demographic inference of effective population size through time obtained with *blockbuster*. From top to bottom, the four rows correspond to successive filtering steps: (i) raw SFS; (ii) SFS after filtering out individuals with low sequencing depth; (iii) SFS after filtering out Z scaffolds; and (iv) SFS after filtering out regions of high heterozygosity and low SNPs density.

For each filtering step, we report the number of individuals (n), the genome length (L′), the total number of segregating sites in the input SFS (S), and the average pairwise difference per site in L′ (π) (Fig. 3). On top of these summary statistics, we inferred the variation of effective population size from the folded SFS with the program “blockbuster” (Omarjee et al. 2026). We set a mutation rate of 5 × 10^−9^ per generation per nucleotide (Smeds et al. 2016) and L′ for the genome length.

Filtering out a few individuals with low coverage (filtering step ii) results in a tenfold increase in S, because the individuals with low coverage have a particularly high amount of missing data. For cases where missing data is more evenly distributed across individuals (eg when sequencing depth is relatively constant across individuals), it may be helpful instead to randomly downsample site by site using tools such as *easySFS* (Anon). Note that after this first filtering step, we see little visual difference in the shape of the raw SFS (Fig. 3), with two clear peaks on the transformed SFS (Achaz 2009; Lapierre et al. 2017) at frequencies 1/2 and 1/4 of the folded SFS. Removing Z scaffolds diminished the height of the 1/4 peak, whereas filtering high heterozygosity regions removes both 1/4 and 1/2. This suggests that these peaks are due, respectively, to hemizygous sex chromosomes and duplicated regions.

Interestingly, it is not possible to properly fit artefactual peaks by varying demographic parameters, but spurious bottlenecks appear when the filtering is not properly done; note that bottlenecks have disappeared in the bottom panel. In the end, the final output shows a reasonable scenario of size variations with six epochs: The Burrowing owl's effective population size increased tenfold (up to an “actual” Ne of 10^6^) about 200 generations ago, in agreement with the “Least Concern” IUCN status (IUCN 2025).

## Conclusions

snpArcher provides a reproducible, end-to-end workflow for producing analysis-ready variant callsets from whole-genome resequencing data, with particular attention to the needs of researchers working with nonmodel organisms. By following this Protocol, users can move from raw sequencing reads to a filtered, joint-called VCF accompanied by an interactive QC dashboard that surfaces common issues such as batch effects, contamination, and cryptic relatedness. The postprocessing steps outlined here, informed by the QC report, help ensure that downstream population genomic analyses are not driven by technical artifacts.

We have focused on practical guidance: installation, configuration, execution, and interpretation, rather than methodological benchmarking, which is covered in the original snpArcher publication (Mirchandani et al. 2024). As variant calling tools and best practices continue to evolve, snpArcher's modular design allows individual components to be updated or replaced without restructuring the entire workflow. The addition of new variant callers such as DeepVariant and bcftools in version 2 reflects this flexibility. Users who encounter limitations or wish to extend the pipeline for their own analyses can contribute custom modules following the project's development guidelines on GitHub.

We hope that this Protocol lowers the barrier to rigorous variant calling for the growing community of researchers undertaking population genomic studies in nonmodel systems, and that the emphasis on quality control encourages careful evaluation of callsets before they are used to draw biological conclusions.

## Data Availability

No new data were generated or analyzed in support of this research.
